# Twin heart with a fused atria and separate ventricles in conjoined twins

**DOI:** 10.4103/0974-2069.74065

**Published:** 2010

**Authors:** Sameer Suresh Ambar, Prabhu C Halkati, Suresh V Patted, ST Yavagal

**Affiliations:** Department of Cardiology, Jawaharlal Nehru Medical College and KLE University, Belgaum, Karnataka, India

**Keywords:** Cardiac malformations in twins, conjoined twins, fused heart

## Abstract

One of the most interesting congenital malformations is that of conjoined twins. We report echocardiographic features of twin heart in dicephalus, tribrachius, dispinous, thoracoomphalopagus twin. It showed two hearts fused at atrial level. Right-sided heart had single atrial chamber with a single ventricle. A single great vessel, aorta, originated from it. Left-sided heart was well developed with two atria and two ventricles. There was a small mid muscular ventricular septal defect and a small patent ductus arteriosus. Great arteries had normal origins.

## INTRODUCTION

One of the most interesting congenital malformations is that of conjoined twins. Conjoined twins are a rare occurrence in medical practice. More commonly known as Siamese twins, this phenomenon is shrouded in mystery and considered a curiosity among the general public. Frequently, the twins are born dead, but there are few cases in which the twins survive. We present cardiac morphology of a case of dicephalus, tribrachius, dispinous, thoracoomphalopagus, a rare type of conjoined twins.

## CASE REPORT

A 26-year-old woman, second gravida, with a normal living female child presented to our hospital at term in the first stage of labor. Her paternal two cousin sisters had given birth to twins. She underwent a prolonged difficult vaginal delivery to deliver live conjoint twins. Placenta was single. Twins were resuscitated and intubated and put on ventilator. The twins survived for 18 hours on ventilator and then died. The ventilatory support was withdrawn as per the parental wish. Autopsy was not performed as the parents refused to give the consent.

**Figure 1:**
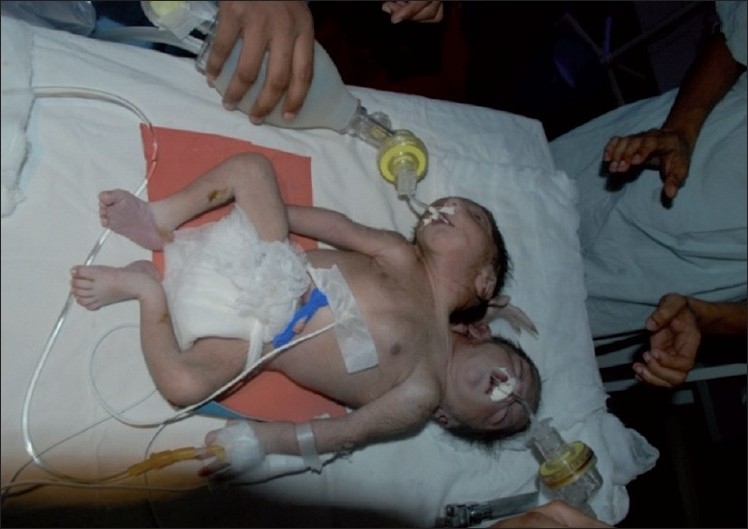
Conjoined twin

**Figure 2:**
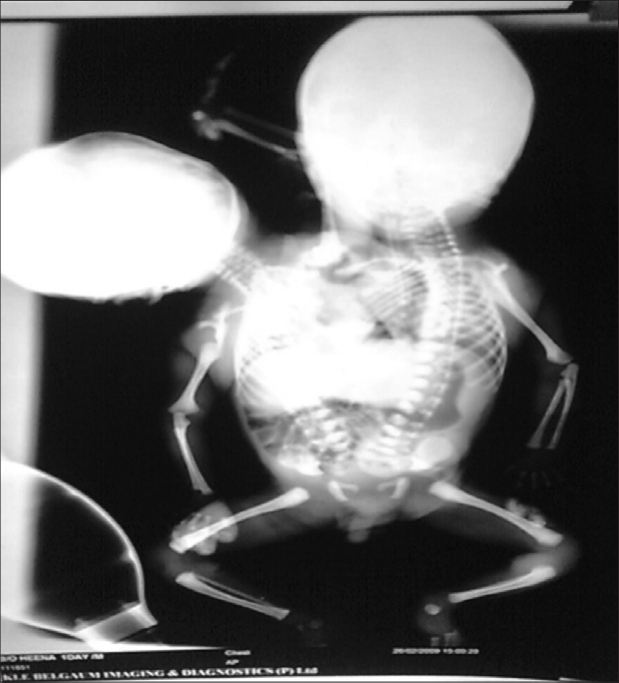
Infantogram showing two heads, two thoraces, two hearts and two vertebral columns, three upper limbs and two lower limbs

External examination showed that the twins were conjoined from the level of thorax downward. The twins had two heads, two upper limbs and a rudimentary arm in between the two heads, shared thorax, abdomen, pelvis and a single male external genitalia and single pair of lower limbs (dicephalus, tribrachius, dispinous, thoracoomphalopagus twins) [Figure 1]. An infantogram revealed that the twins had two heads, two vertebral columns, three upper limbs and a shared abdomen, pelvis and a single pair of lower limbs [Figure 2]. Upper limbs had unequal saturations, right upper limb had a saturation of 50% and left upper and lower limbs had a saturation of 90%. Ultrasonography of the abdomen revealed a shared liver, a single spleen on the left side, single pair of kidneys, two vertebral columns, bilateral inferior vena cavae and a single left abdominal aorta.

**Figure 3:**
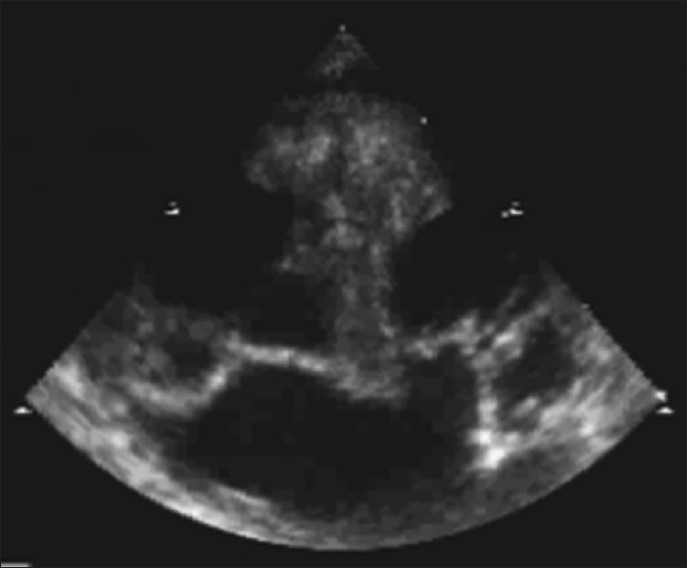
Echo showing two hearts fused at atrial level. Right-sided heart had single atrial chamber communicating with a single ventricle through single atrioventricular valve and left-side heart communicated with two left-sided ventricles

**Figure 4:**
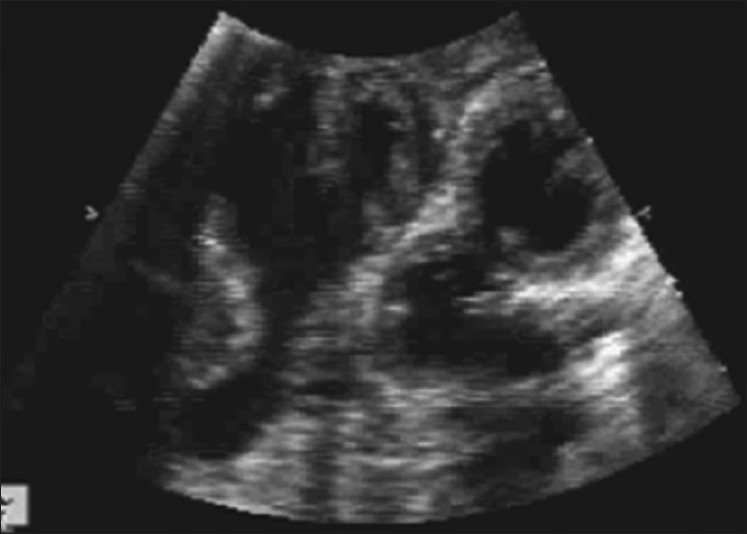
Right-sided heart single ventricle had large ventricular septal defect. A single great vessel, aorta, originated from the ventricle. Two ventricles of left-sided heart are also seen

Echocardiography showed midline liver, two vertebral columns with two inferior vena cavae (IVC) on either sides and a left-sided descending aorta. There were two hearts fused at the atrial level [Figure 3]. Right-sided heart had single atrial chamber communicating with a single ventricle through single atrioventricular valve [Figures 3 and 4]. The atrium received right-sided IVC without any drainage of pulmonary veins. A single great vessel, aorta, originated from it, forming a right aortic arch. Pulmonary artery was not seen in right-sided heart.

**Figure 5:**
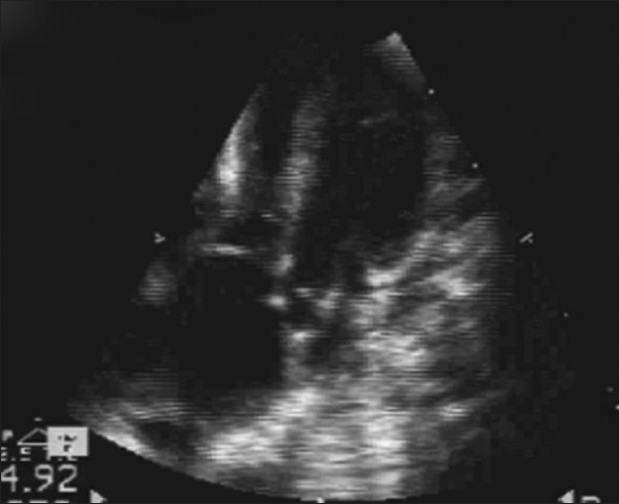
Left-sided heart with two atria, two atrioventricular valves and two ventricles

**Figure 6:**
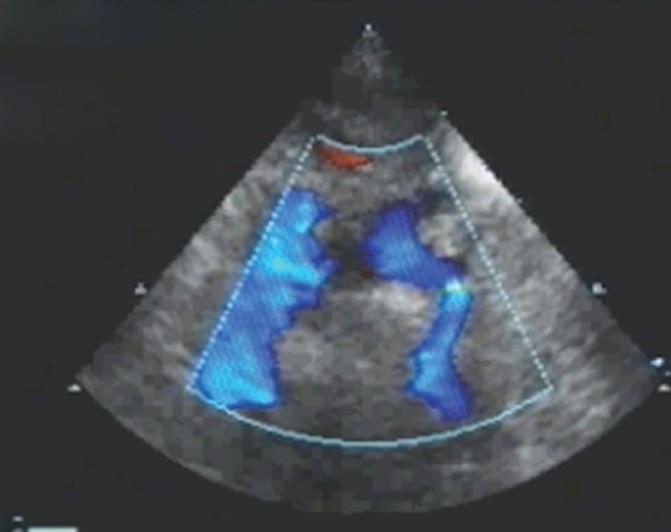
Hypoplastic right aortic arch from right heart

Left-sided heart was well developed with two atria, two atrioventricular valves and two ventricles [Figure 5]. Right atrium received left-sided IVC and left atrium received two pulmonary veins. Right atrium was communicating with the single atrium of right-sided heart. Interatrial septum was intact, while the mid muscular interventricular septum had small ventricular septal defect with left to right shunt. Aorta and pulmonary arteries had normal origins and were normally related. There was a small patent ductus arteriosus with left to right shunt. Aorta continued as a left-sided arch with normal branches [Figure 6].

## DISCUSSION

The incidence of conjoined twins is reported to be in the range of 1 in 50,000 to 1 in 100,000 live births;^1^ but as 60% are stillborn or die shortly after birth, the true incidence is around 1 in 200,000 live births.[2] Girls predominate in the ratio of 3:1.[1,2]

Twins are classified according to the major site of union, to which the ending “pagus” is added, meaning “fixed”.[2]

1. thoracopagus: joined at the thoracic level; the most common type accounting for 40% of cases,
2. omphalopagus: joined at the abdomen, often including the lower thorax; accounting for 32% of cases,
3. craniopagus: joined at the head, accounting for 6% of cases,
4. pyopagus (sacral fusion), ischiopagus and
5. parapagus used to describe twins fused extensively side to side.

Cardiac abnormalities are present in any type of fusion. Thoracopagus twins have the highest incidence of cardiac anomalies with a 90% incidence of shared pericardium[3] and major myocardial connections in 75% of cases.[4] The extent of cardiac fusion and intracardiac anatomy (ICA) in conjoined twins determines not only the potential for surgical separation but also the long-term survival. Surgical separation is rarely feasible in complex fused hearts.[5,6] The cardiac anomalies in twins are classified as

1. group A: separate hearts, separate pericardium,
2. group B: separate hearts, common pericardium,
3. group C: fused atria, separate ventricles and
4. group D: atrial and ventricular fusion (all thoracopagus).

In our case, the cardiac anomaly was of group C type. This case represents a complex anatomy and physiology in a twin. A conjoined twin is frequently the mirror image of its partner and this is particularly true in dicephalus twins. In our case, the right-sided heart had multiple anomalies and the left-sided heart was nearly normal. The circulation physiology was complex. The single, fused atrium of right-sided heart received deoxygenated blood from right-sided inferior vena cava. There was no pulmonary venous drainage to it. This atrium was fused with right atrium of left-sided heart which had intact interatrial septum. The right atrium of the left-sided heart received deoxygenated blood from left-sided IVC. The deoxygenated blood from right-sided IVC and from left-sided vena cava through fused atria circulated in right-sided twin through single atrium, single ventricle and single great vessel, aorta, leading to cyanosis in right upper limb. The left-sided heart was well developed. Pulmonary veins were draining from the left lung to left atrium with intact interatrial septum in the left-sided heart. The cardiac circulation was normal except for a small ventricular septal defect and small patent ductus arteriosus with left to right shunt. The left upper limb had normal saturation. The liver was single and there was one spleen on the left side. The right stomach was atrophic and the intestines joined distal to the duodenum, at the level of Meckel’s diverticulum. Diaphragmatic hernia was present. Such complex cardiovascular anomalies are not amenable to surgical correction.

The present case highlights morphologic features of an antenatally undiagnosed dicephalus, tribrachius, dispinous, thoracoomphalopagus twins, which is a rare form of conjoined twins. It shows a rare anatomy and physiology of human systems which was incompatible with survival. Early prenatal diagnosis and precise characterization of conjoined twins are essential for optimal interventional and postnatal management. Successful separation of thoracopagus twins in cases of conjoined heart is difficult. There has been only a single report of successful separation of a conjoined heart and in this instance it was conjoined atria.[7] In a series of 13 surgeries on conjoined twins in The Children’s Hospital of Philadelphia, none of the patients with complex fused hearts survived even after sacrificing one of the twins.[7] Cases of thoracophagus conjoined twins with normal separate hearts, common pericardium, anomalous systemic venous drainage, fused atria and atrial septal defects have been successfully separated.
